# Inhibiting inducible miR-223 further reduces viable cells in human cancer cell lines MCF-7 and PC3 treated by celastrol

**DOI:** 10.1186/s12885-015-1909-2

**Published:** 2015-11-09

**Authors:** Lu Cao, Xue Zhang, Fanfan Cao, Ying Wang, Yufan Shen, Chunxin Yang, Georges Uzan, Bin Peng, Denghai Zhang

**Affiliations:** 1 Department of Clinical Laboratory Diagnostics, Postgraduate Education College, Ningxia Medical University, Yinchuan, 750004 China; 2 Sino-French Cooperative Central Lab, Shanghai Gongli Hospital, the Second Military Medical University, 207 Ju Ye Road, Pudong New District, Shanghai, 200135 China; 3 Pharmaceutical Department, Zhong Shan Hospital, Shanghai Fudan University, 136 Yi Xue Yuan Road, Shanghai, 200032 China; 4 U972, Inserm, Bâtiment Lavoisier, Hôpital Paul Brousse, 12 Avenue Paul Vaillant Couturier, 94807 Villejuif Cedex, France

**Keywords:** miR-223, Celastrol, Anti-cancer, MCF-7, PC3, NF-κB, mTOR, HSP70

## Abstract

**Background:**

Celastrol is a novel anti-tumor agent. Ways to further enhance this effect of celastrol has attracted much research attention.

**Methods and Results:**

Here, we report that celastrol treatment can elevate miR-223 in human breast cancer cell line MCF-7 and prostate cancer PC3. Down-regulating miR-223 could increase the number of viable cells, yet it further reduced viable cells in samples that were treated by celastrol; up-regulation of miR-223 displayed opposite effects. Celastrol’s miR-223 induction might be due to NF-κB inhibition and transient mTOR activation: these two events occurred prior to miR-223 elevation in celastrol-treated cells. NF-κB inhibitor, like celastrol, could induce miR-223; the induction of miR-223 by NF-κB inhibitor or celastrol was reduced by the use of mTOR inhibitor. Finally and interestingly, miR-223 also could affect NF-κB and mTOR and the effects were different between cells treated or not treated with celastrol, thus providing an explanation for differing effects of miR-223 alteration on cellular viability in the presence of celastrol or not.

**Conclusions:**

For the first time, we disclose that celastrol could induce miR-223 in breast and prostate cancer cells, and that inhibiting miR-223 could further reduce the living cells in celastrol-treated cancer cell lines. We thus provide a novel way to increase celastrol’s anti-cancer effects.

## Background

Celastrol is a natural compound extracted from the plant *triperygium wilfordii* Hook F, which has been used in anti-inflammation and anti-cancer treatments in Chinese folk medicine for many years. Celastrol has proven effective in treating a variety of cancers [1], including those arising from breast [2, 3], prostate [4], lung [5], liver [6], digestive tract [7], skin [8], and leukemia cells [9], among others. Celastrol is a promising anti-cancer agent and has attracted the attention of researchers.

It is commonly accepted that cancer cells surviving chemotherapy will become resistant to re-use of the same drugs and cause cancer relapse. It follows that improving the effects of anti-cancer agents could reduce or delay cancer re-occurrence. In line with this notion, we and others have worked on new ways to enhance celastrol’s anti-cancer effects, especially by focusing on heat shock response (HSR). Celastrol has been found to induce heat shock response in multiple cancer cell lines because of its activation of heat shock factor-1 (HSF-1) [10]. For example, Matokanovic et al. used siRNA to reduce HSP70 levels, thus increasing celastrol’s anti-cancer ability [11]. Our research found that a peptide deformylase inhibitor, actinonin, could reduce celastrol-induced HSP70 and increase celastrol’s anti-proliferation effects [12]. It is reasonable to think that there might be other treatment-caused responses that affect celastrol’s anti-cancer effects. To identify these might provide a new way to enhance celastrol’s role as an anti-cancer agent.

It has been reported that miR-223 influences the survival ability of various cancer cells [13]. Yang et al. found that miR-223 promoted the invasion of breast cancer cells via the Mef2c-β-catenin pathway [14], while Pinatel et al. reported that overexpressing miR-223 decreased migration, increased cell death in anoikis conditions and augmented sensitivity to chemotherapy, but had no effect on adhesion and proliferation [15]. miR-223 is also reported to promote the biological behavior of prostate cancer [16], contribute to gastric cancer cell proliferation and migration [17], and function as an oncogene in human colorectal cancer cells [18]. Recently, we found that celastrol could induce miR-223 in human hepatoma cells (unpublished).

Therefore, whether or not celastrol-caused miR-223 elevation affects celastrol’s anti-cancer action, and if so, why, are questions worth addressing. To do so, we first observed miR-223 alterations caused by celastrol in human breast cancer line MCF-7 and prostate cancer line PC3 (two of the most common types of cancer and the two cancer types most often used in celastrol studies), as well as the effects of manipulating miR-223 on celastrol’s ability to reduce the number of living cells. Then, we investigated the possible reason for celastrol’s miR-223 induction by focusing on how altering NF-κB affects miR-223 expression, since celastrol is a known NF-κB regulator [19–21], and NF-κB reportedly regulates miR-223 [22]. In addition, in pre-experimental trials, we found that NF-κB activity affected and was linked to mTOR activity and HSP70 levels. Therefore, the effects of altering mTOR and HSP70 on miR-223 expression were also investigated. Finally, we tried to find the possible molecular basis by which miR-223 alterations affected cellular viability in cells treated or not treated with celastrol. Again, we focused on NF-κB, mTOR, and HSP70, since these three molecules are widely reported as related to celastrol’s anti-tumor effects [10, 23–26]. Importantly, miR-223 could regulate NF-κB [27], mTOR [28, 29], and members of the heat shock protein family [28].

## Methods

### Reagents and drugs

Dimethyl sulfoxide (DMSO) was purchased from Sigma (St. Louis, MO). NF-κB inhibitor (PDTC) and mTOR inhibitor (Ku-0063794) were obtained from Roche (Mannheim, Germany). Carboxyfluorescein diacetate, succinimidyle ester (CFSE) was from Molecular Probe (Eugene, OR) and 7-Amino-actinomycin D (7-AAD) was purchased from Anaspec (San Jose, CA). Protein Extraction Kit, BCA protein assay reagent kit and Beyo ECL Plus for western blot were purchased from Beyotime Biotechnology (Jiangsu, China). Anti-phospho-HSF-1 (Ser326), anti-phospho-mTOR (Ser2481), and anti-mTOR were purchased from Epitomics (CA). Anti-β-action, anti-HSP70, anti-phospho-NF-κB (Ser536) and horseradish peroxidase (HRP)-labeled secondary antibodies were purchased from Cell Signaling Technology (MA).

Celastrol was extracted as previously reported by us [30, 31]: briefly, the air-dried root bark of triperygium wilfordii Hook F (from Fujian Province, China) was powdered and extracted in refluxing n-hexane, the extract was chromatographed on silica gel and eluted with gradient n-hexane/acetone. The celastrol-containing fraction (red color) was collected, evaporated, and recrystallized with acetone to produce celastrol (needle red crystal). The purity of the obtained celastrol was over 99.0 %, as determined by high-performance liquid chromatography (Agilent 1200, Santa Clara, CA; celastrol standard was from Sigma). The celastrol was dissolved in DMSO at 50 mM. The celastrol solution was stored at -20 °C and used within 3 months of preparation. The stored solution was further diluted with RPMI 1640 medium or DMEM to a proper lower concentration immediately prior to experiments.

### Cell culture and treatment by Celastrol, PDTC and Ku-0063794

Breast cancer cell line MCF-7, prostate cancer cell line PC3 and leukemic cell lines U937 used in this study were all obtained from the Shanghai Cell Bank of the Chinese Academy of Sciences (Shanghai, China). Cells were maintained in RPMI 1640 or DMEM supplemented with 10 % FBS, 100 IU/ml penicillin and 100 μg/ml streptomycin (all from PAA Laboratories, Linz, Austria), and cultured in a humidified 5 % CO_2_ atmosphere in an incubator at 37 °C. Exponentially growing cells were used for experiments. Cells were seeded into 24-well (2 × 10^5^ cells) or 6-well (8 × 10^5^ cells) culture plates followed by exposure to the indicated doses of celastrol, or PDTC (20 μM), or Ku-0063794 (3 μM) or the combination of these agents for the indicated times. The culture medium with DMSO (vehicle) served as control. Each experiment was repeated at least three times.

### Cell transfection

2 × 10^5^ cells were seeded in 24-well plates or 8 × 10^5^ cells were seeded in 6-well plates for 1 day before transfection. MiR-223-Down antagomir, miR-223 mimics, or siRNA for HSP70 gene or their controls were transfected into cells by siRNA-mate (GenePharma Shanghai, China), according to the manufacturer recommendations. The transfection was terminated by PBS washing after 24 h and the cells were used for subsequent experiments as indicated.

### Cell counting by flow cytometry (FCM)

After receiving different treatments, cells were collected and washed with PBS. Cell numbers were accurately quantified by flow cytometry, based on a single-tube platform with self-made cell-beads as internal controls, a method originally reported by Harrison et al. [32] and modified by us [33]. Briefly, samples were collected and then a known number of self-made CFSE-containing cell-beads added. The cell-beads contained green fluorescence, made by incubation of U937 cells with CFSE. Before analysis by FACScalibur flow cytometer (Becton-Dickinson, CA), 7-AAD was added with a final concentration of 1 μg/ml for separating dead cells. The number of vital (or dead) cells was calculated using the following equation:

$$ \begin{array}{l}\begin{array}{l}\mathrm{Number}\ \mathrm{of}\ \mathrm{vital}\ \left(\mathrm{dead}\right)\ \mathrm{cell}\mathrm{s} = \kern0.5em \Big[\mathrm{number}\ \mathrm{of}\ \mathrm{vital}\ \left(\mathrm{dead}\right)\ \mathrm{cell}\mathrm{s}\ \mathrm{detected}/\hfill \\ {}\mathrm{number}\ \mathrm{of}\ \mathrm{cell}\hbox{-} \mathrm{beads}\ \mathrm{detected}\Big]\hfill \end{array}\\ {}\kern21.5em  \times \mathrm{number}\ \mathrm{of}\ \mathrm{cell}\hbox{-} \mathrm{beads}\ \mathrm{input}\end{array} $$

### RNA isolation and Quantitative real-time PCR (qRT - PCR)

Total RNA was extracted from MCF-7 and PC3 cells using an RNA extraction kit (Fastagen Biotechnology, Shanghai, China) according to the manufacturer’s protocols. One microgram of total RNA was reverse transcribed, using the ReverTra Ace qPCR-RT Master mix kit (Toyobo, Qsaka,Japan) and reverse-transcription primers specific to the miRNAs of interest (GenePharma). The reaction was performed on Applied Biosystems (CA), at 37 °C for 15 min, 50 °C for 5 min, 98 °C for 5 min, and stopped at 4 °C. The obtained miRNA-specific cDNA was amplified on an Agilent MX3000P machine (CA), using the Realtime PCR master mix (Toyobo); with processing at 95 °C for 30 s, followed by 40 cycles at 95 °C for 5 s, 55 °C for 5 s, and 72 °C for 30 s. U6 small nuclear RNA was used as an endogenous control. The fold change in the level of detected miRNA was calculated using the following equation:

$$ \Delta \mathrm{C}\mathrm{t} = \mathrm{C}\mathrm{t}\ \mathrm{miRNA}\ \hbox{--}\ \mathrm{C}\mathrm{t}\ \mathrm{U}6 $$

$$ {{\mathrm{Relative}\ \mathrm{Expression}\ \mathrm{Ratio} = 2}^{\hbox{-}}}^{{}^{\Delta}\mathrm{C}\mathrm{t}} $$

### Western blot

MCF-7 and PC3 cells were incubated in lysis buffer and cleared by centrifugation at 13,000 × g for 15 min. The extraction of cytoplasmic and nuclear protein was performed according to product manufacturer instructions. The BCA protein assay reagent kit determined protein concentrations. Proteins were separated by 10 % SDS-polyacrylamide gels and then transferred to polyvinylidenedifluoride (PVDF) membranes (Millipore, Bedford, MA). Membranes were probed with antibodies against the proteins of interest. Detection was accomplished using corresponding HRP-conjugated secondary antibodies followed by development with Beyo ECL Plus. Pictures were captured and band densities measured by G: BOX iChemi XR (Syngene Inc., UK).

### Statistical analysis

All experiments were repeated at least three times. Data are presented as the mean ± SD. The data from multiple groups were analyzed with a one-way analysis of variance. Data from two groups were compared by t-tests. A value of *P* < 0.05 was considered significant.

## Results

### Celastrol reduced viable cells and induced miR-223 expression in MCF-7 and PC3 in time- and dose-dependent ways

**Fig. 1:**
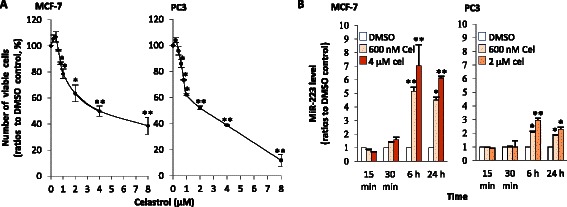
Effects of celastrol on number of living cells and miR-223 in two cell lines. **a** Celastrol’s effects on viable cell numbers. MCF-7 or PC3 cells were cultured with different doses of celastrol (with solvent DMSO as control) for 24 h. Then the number of viable cells was quantified by flow cytometry with one-platform protocol, as detailed in Methods. **b** Inducing miR-223 with celastrol. After incubation with celastrol at the indicated doses for the indicated time, the cells’ total RNA was extracted and reverse transcription was performed. Then miR-223 levels were determined by quantitative PCR with specific primers. Cel represents celastrol. The tests were repeated three times. Data is presented as mean ± SD; statistics were determined between the celastrol-treated group and DMSO, * indicates *P* < 0.05, while ** indicates *P* < 0.01

We first observed the dose-effects of celastrol on the number of living cells in human breast cancer cell line MCF-7 and prostate cancer cell line PC3. As shown in Fig. 1a, celastrol treatment for 24 h reduced viable cells in MCF-7 and PC3, the IC50 for MCF-7 and PC3 cells were about 4 μM and 2 μM, respectively (Fig. 1a), these doses were used for the two cell lines for most of the following experiments.

We found that after 6 h treatment, celastrol could significantly induce miR-223 expression in the two cell lines. MCF-7’s response was more pronounced than the PC3 response. Celastrol-caused induction still existed at 24 h. miR-223 induction was dose-dependent in that higher dosages resulted in higher miR-223 levels (Fig. 1b).

### Down-regulating or overexpressing miR-223 increased or decreased the loss of viable cells in celastrol-treated samples, respectively

Since celastrol could induce miR-223 expression, and this small RNA is reported to affect the viability of cancer cells, including MCF-7 and PC3 [13–16], we were interested in whether regulating miR-223 levels affected celastrol’s anti-cancer ability. To address this issue, we inhibited or over-expressed miR-223 and observed the effects of such manipulation on celastrol’s action.

**Fig. 2:**
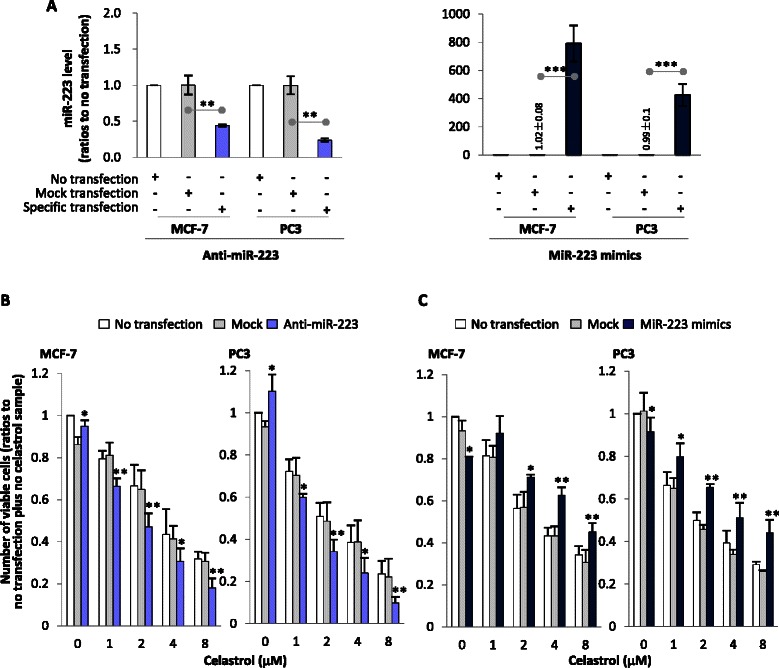
Effects of regulating miR-223 on viable cell numbers. **a** Regulating miR-223 by transfection. Cells were transfected with miR-223-Down antagomir (shortened to anti-miR-223) or miR-223 mimics or corresponding mock agent (mock transfection) for 24 h, then the miR-223 levels were determined by PCR to confirm if transfection was successful. **b** and **c** miR-223’s effects on viable cell numbers. After cells were transfected with anti-miR-223 (**b**) or miR-223 mimics (**c**) for 24 h, the cells were washed with PBS and then cultured with celastrol at different doses or with DMSO for 60 h. Cell numbers were determined by flow cytometry. Tests were repeated three times, data is presented as mean ± SD, statistics were determined between specific transfection and mock transfection, or as demonstrated in figures. * indicates *P* < 0.05, while ** and *** indicate *P* < 0.01 and *P* < 0.001, respectively

After 24 h transfection with miR-223-Down antagomir or miR-223 mimics, miR-223 was inhibited or elevated, respectively (Fig. 2a). The results of culture-transfected cells after 60 h showed that down-regulating or overexpressing miR-223 alone could increase or decrease viable cells in MCF-7 and PC3 when compared to controls (mock transfection) (Fig. 2b, c). However, cells receiving miR-223-Down antagomir transfection had lower numbers of viable cells in the presence of celastrol, when compared to those without transfection or mock transfection (Fig. 2b). On the contrary, cells with miR-223 mimic transfection reduced the loss of viable cells in presence of celastrol, when compared to no transfection or mock transfection (Fig. 2c). These results indicate that blocking miR-223 elevation could further reduce living cells in samples treated with celastrol.

### Celastrol affected mTOR, NF-κB, and HSF-1 activation before inducing miR-223

We were then interested in why celastrol could induce miR-223. Since celastrol is a strong NF-κB regulator [19–21], and NF-κB reportedly regulates miR-223 [22], we thought that regulating NF-κB might be involved in miR-223 induction. Additionally, our pre-experimental trial showed that changing NF-κB could result in, and was thus linked to alterations in mTOR activity and HSP70 levels in MCF-7 and PC3 (data not shown). These two molecules might also play roles in celastrol’s miR-223 regulation. Nevertheless, if a molecular alteration is the cause behind celastrol’s miR-223 induction, it should meet two requirements, (1) following celastrol treatment, this alteration must happen before miR-223 induction and (2) this alteration itself could regulate miR-223 in our system.

**Fig. 3:**
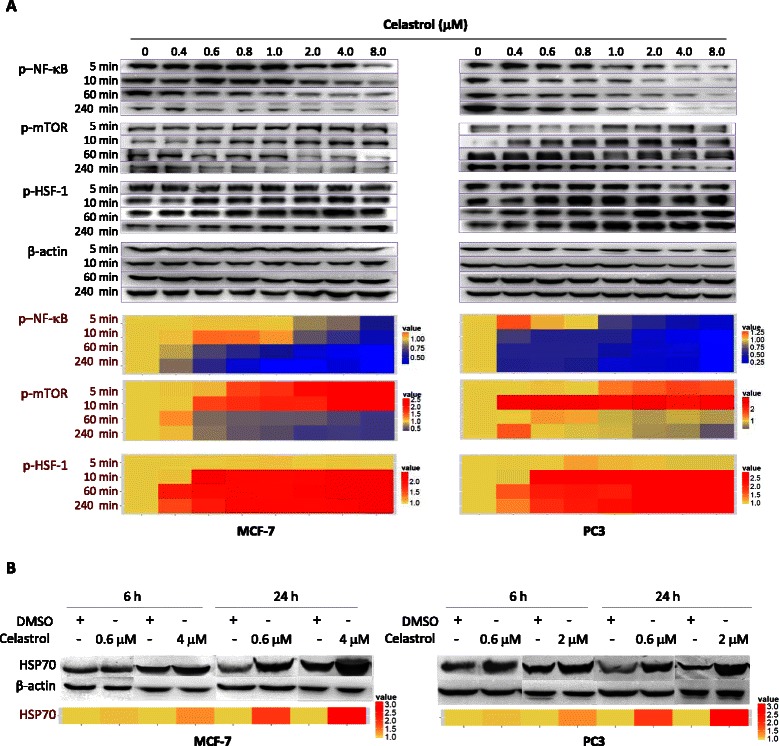
Celastrol’s effects on multiple molecules. MCF-7 or PC3 cells were treated with different concentrations of celastrol for the indicated time, and phosphorylated NF-κB (p-NF-κB), p-mTOR, and p-HSF-1 (panel **a**), as well as total HSP70 (panel **b**), were detected by Western blot with the corresponding antibody. The band density was measured, and the density of specific proteins of one sample was adjusted by the density of β-actin in the same sample. The relative expression of one protein of interest (X) in the test sample was found by the algorithm: (adjusted density of X in test sample)/(adjusted density of X in control sample), with the relative expression of X protein in the control set as 1. The mean of relative expressions of all specific proteins in different treatments is displayed by heat map, which was created by ggplot2 package of R language (https://www.r-project.org/). The experiments were repeated three times

Therefore, we first observed whether celastrol-caused alterations in NF-κB, mTOR and HSP70 occurred, and if they preceded miR-223 elevation (which were obvious at 6 h after celastrol treatment). As shown in Fig. 3a, reductions in NF-κB phosphorylation, indicating NF-κB inhibition, were seen as early as 5 min after celastrol addition. This change remained at least to 6 h, the end point of observations in our study. At 5 min after celastrol loading, mTOR phosphorylation was increased, indicating the cells had increased mTOR activation; however, by 1 h after celastrol treatment, mTOR activation began to be inhibited (Fig. 3a). Celastrol-caused mTOR inhibition has been reported [23, 26], yet, for the first time we found that prior to inhibition there was an early and transient increase in mTOR activation. HSF-1 activations were seen at 10 min after celastrol treatment (Fig. 3a). HSF-1 activation is reported as important to celastrol-caused heat shock response, a hallmark of which is HSP70 elevation [10]. In agreement with such findings, HSP70 elevation was observed at 6 h after celastrol addition (Fig. 3b), a time-point coinciding with miR-223 elevation (Fig. 1b). These alterations in mTOR, NF-κB, and HSF-1 activation, as well as HSP70 induction, were not only time-dependent, but also dose-dependent, in that higher doses prompted stronger responses.

The above data shows that, NF-κB inhibition and transient mTOR activation, but not HSP70 elevation, occurred before miR-223 elevation and thus might be the cause of miR-223 elevation in celastrol-treated cells.

### Inducing miR-223 by celastrol was related to NF-κB inhibition and mTOR activation

**Fig. 4:**
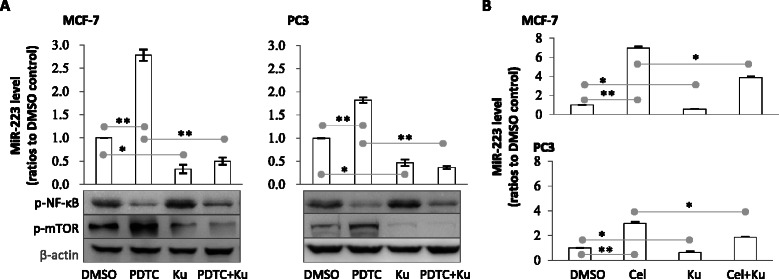
Effects of NF-κB and/or mTOR acidity on miR-223 expression. **a** Effects of NF-κB inhibitor and/or mTOR inhibitor on miR-223. MCF-7 or PC3 cells were treated with NF-κB inhibitor (PDTC) and/or mTOR inhibitor Ku-0063794 (abbreviated as Ku) for 1 or 6 h, then, the RNA (6 h samples) was extracted and miR-223 determined by quantitative reverse-transcription PCR. Protein (for 1 h) was extracted and phosphorylated NF-κB and mTOR were determined by Western blot. **b** Effects of mTOR inhibitor on celastrol-induced miR-223 expression. Cells were treated with celastrol and/or mTOR inhibitor for 6 h, then the miR-223 was detected. Cel represents celastrol. Tests were repeated three times. Data are presented as mean ± SD; * indicates *P* < 0.05, while ** indicates *P* < 0.01

Then, we observed the effects of altering NF-κB or mTOR activity on miR-223 expression. We found that NF-κB inhibitor could induce miR-223, while mTOR inhibitor down-regulated miR-223 (Fig. 4a). When the two inhibitors were used together, miR-223 elevation was inhibited (Fig. 4a). mTOR inhibitor also reduced celastrol-caused miR-223 elevation (Fig. 4b). This suggests that mTOR activation was necessary for miR-223 induction. To evidence this notion, we found that the NF-κB inhibitor that elevated miR-223 could also increase mTOR activation (Fig. 4a). These findings suggest that celastrol’s miR-223 induction was related to NF-κB inhibition and transient mTOR activation (as shown in Fig. 3a).

We also used siRNA transfection to knock down HSP70 and found that the cells with lowered HSP70 had decreased miR-223 (data not shown), suggesting that celastrol-induced HSP70 elevation might contribute to miR-223 maintenance, though this effect would occur at a later stage, such as more than 6 h after celastrol treatment.

### miR-223 affected cellular viability-related NF-κB, mTOR activity, and HSP70 level

The final step in this work was to try to find the possible molecular basis for miR-223’s effects on cellular viability in samples treated or not treated with celastrol. Again, we focused on the relationship between miR-223 and NF-κB, mTOR, and HSP70, but in reverse. That is to say, we looked into the possible effects of miR-223 on these molecules, since these molecules, as mentioned above, reportedly affect celastrol’s anti-tumor ability, and also because miR-223 has been confirmed as regulating these molecules [27–29]. If a molecular alteration is in fact the basis for miR-223’s effects on celastrol’s ability to reduce viable cells, this molecular alteration should affect cellular viability in cells treated with celastrol in addition to being regulated by miR-223 alterations.

**Fig. 5:**
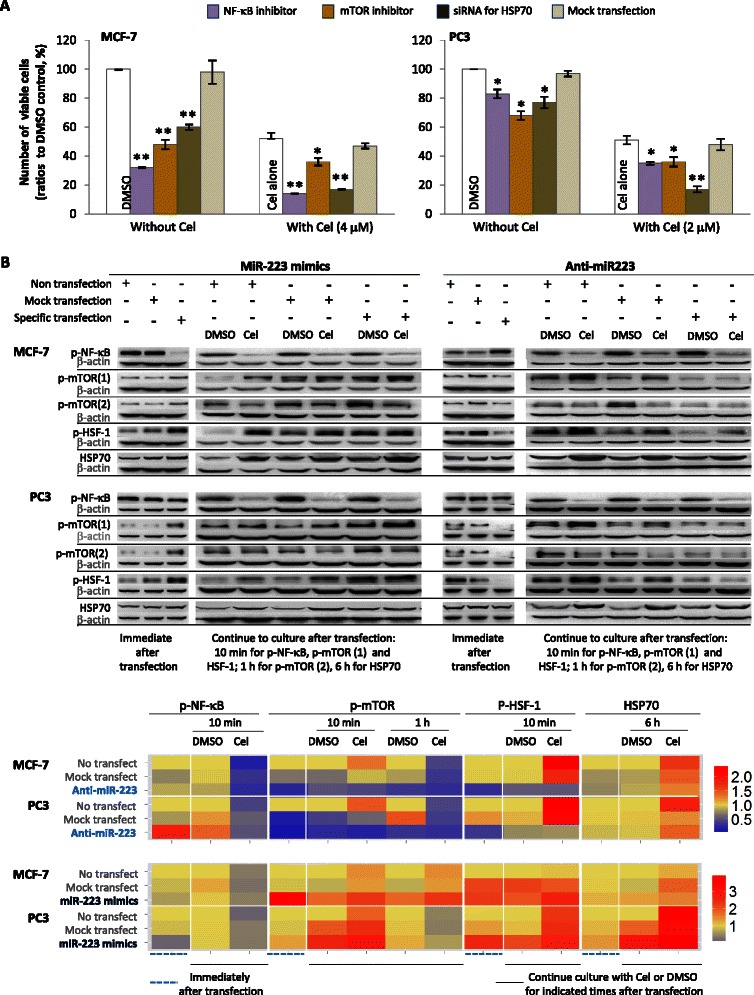
Effects of on multiple molecules on cellular viability and miR-223 regulation of these molecules. **a** Effects of multiple molecules on cellular viability. The cells were pre-treated with NF-κB inhibitor (PDTC) or mTOR inhibitor Ku-0063794 (abbreviated as Ku) for 1 h, or transfected with siRNA specific to *hsp70* gene or non-specific siRNA (mock) for 24 h and subsequently washed to stop transfection. Cells were then treated with celastrol (4 μM for MCF-7 and 2 μM for PC3) or DMSO for 24 h, before the numbers of viable cells were quantified with flow cytometry. **b** Effects of miR-223 alteration on multiple molecules. Cells were transfected with miR-223-Down antagomir (shortened to anti-miR-223) or miR-223 mimics or corresponding mock agent (mock transfection) for 24 h, then the cells were washed to stop transfections. Some of the cells were used immediately to extract protein; the remaining ones were used for culture with celastrol (4 μM for MCF-7 and 2 μM for PC3) or DMSO for the indicated time, before proteins were extracted. The obtained proteins were used to detect phosphorylated NF-κB (p-NF-κB), p-mTOR, and p-HSF-1, as well as whole levels of HSP70 by Western blot, with respective primary antibodies and corresponding secondary antibodies being used. The mean of relative expressions of specific proteins in different treatments is displayed by heat map (detailed in the legend of Fig. 3). Tests were repeated three times. Cel represents celastrol. Data in A panel are presented as mean ± SD, statistics were determined between different treatments (without celastrol) and DMSO control, or between different treatments (plus celastrol) and celastrol alone * indicates *P* < 0.05, while ** indicates *P* < 0.01

We therefore first observed the effects of NF-κB, mTOR, or HSP70 activation on cell viability. As shown in Fig. 5a, knocking down HSP70 or use of either NF-κB or mTOR inhibitor alone could reduce the numbers of viable cells in MCF-7 or PC3. In samples treated by celastrol, any one of these manipulations could reduce the number of living cells further. Thus, NF-κB and mTOR activation, as well as HSP70 elevation, were important for cellular survival in samples treated or not by celastrol.

We then observed the effects of miR-223 on NF-κB, mTOR and HSP70 in cells treated or not by celastrol. As shown in Fig. 5b, down-regulating miR-223 alone could activate NF-κB (in favor of survival), yet inhibited mTOR and HSF-1 activity and decreased HSP70 level (prone to reducing survival). These effects are contrary, but perhaps the effects of NF-κB alteration are dominant (a hypothesis adjusted in the Discussion section), making the final outcome of miR-223 down-regulation an increase in viable cells (as presented in above Fig. 2b). The observed decrease in viable cells from up-regulating miR-223 (Fig. 2b) is also explainable through NF-κB dominance: this manipulation inhibits NF-κB while activating mTOR and HSF-1 and elevating HSP70 (Fig. 5b).

Interestingly and strangely, the above data showed that miR-223 down-regulation could activate NF-κB in cells not treated with celastrol, however, when celastrol was used, cells with knocked-down miR-223 showed lower NF-κB and mTOR activity than cells with mock transfection. This indicates that down-regulating miR-223 enhanced celastrol-caused NF-κB and mTOR activity inhibition (both prone to reducing survival) (Fig. 5b). This provides an explanation for the finding that miR-223 down-regulation increased loss of living cells in celastrol-treated samples (Fig. 2b), rather than promoting survival, as when celastrol was not used (Fig. 2b). Up-regulating miR-223 ameliorated celastrol-caused NF-κB and mTOR inhibition (Fig. 5b), a finding consistent with the result that miR-223 overexpression reduced the loss of living cells in celastrol-treated samples (Fig. 2b). Altering miR-223, however, showed inconsistent effects on celastrol-induced HSP70 elevation in the two cell lines (Fig. 5b).

## Discussion

In this work, we found that both non-toxic and toxic doses of celastrol could induce miR-223 expression in human cancer cell lines MCF-7 and PC3, and that miR-223 down-regulation further reduced the number of living cells in these two cancer lines treated by celastrol.

One interesting finding of our work is that celastrol could induce miR-223 in MCF-1 and PC3. Such an action was significant at 6 h after celastrol loading, and the effect increased with dose. Results were cell-context dependent, as MCF-7 showed a stronger response than PC3. To support that celastrol might affect microRNA, it was recently reported that celastrol decreased miR-224 and miR-21 [7, 34]. Celastrol’s induction of miR-223 helps to explain the mechanisms behind some of celastrol’s other known actions, such as celastrol’s newly identified potential in anti-obesity applications, since miR-223 is not only a major regulator of cholesterol (miR-223 can reduce cholesterol) [35], but also an insulin sensitivity enhancer [36]. miR-223 induction is also consistent with celastrol’s efficacy in anti-inflammation applications [37, 38].

Another finding is that miR-223 affected celastrol’s ability to reduce numbers of viable cancer cells. We confirmed that when miR-223 was inhibited in MCF-7 and PC3 cells treated by celastrol, the numbers of living cells could be further reduced, while down-regulating miR-223 alone increased the number of living cells. Over-expression of miR-223 had the opposite effect on viable cell numbers when compared to miR-223 down-regulation, either when used alone or when in combination with celastrol. Celastrol’s anti-proliferation effects were less pronounced in MCF-7 than PC3. This difference may be attributed to MCF-7’s higher miR-223 levels induced by celastrol. The effects of miR-223 on breast cancer are harder to explain due to inconsistent research data, with some studies showing promoting effects and others showing suppression [14, 15]. The reason for this discrepancy requires further investigation. Wei et al. recently reported that miR-223 promoted prostate cancer behavior [16], yet this was different from our results. One explanation for the difference is that they did not wash away the transfection reagents when performing their analysis, while we did. Transfection reagents are often toxic to cells, and might affect outcomes. Indeed, celastrol is a toxic agent, and our results showed that in presence of celastrol, miR-223 overexpression promoted survival. Nevertheless, our work suggests that simultaneous inhibition of miR-223 induction could be a novel way to increase celastrol’s anti-cancer ability, at least in breast or prostate cancer.

Third, we found that NF-κB, mTOR, or HSP70 could regulate miR-223. This offers a possible explanation for celastrol’s miR-223 induction. Based on the following facts, we thought that celastrol-induced miR-223 might be related to NF-κB inhibition and rely on transient mTOR activation. One: NF-κB inhibition and mTOR activation (both observable at 5 min after celastrol treatment, though mTOR was inhibited by 1 h) preceded miR-223 elevation (at 6 h). Two: like celastrol, NF-κB inhibitor PDTC could induce miR-223. This effect was accompanied by mTOR activation. Three: mTOR inhibitor could reduce celastrol- or NF-κB inhibitor-induced miR-223 elevation. Kumar et al. reported that in combination with Notch, NF-κB activation, rather than inhibition, elevated miR-223 [22]. This difference in results might be cell-type specific. To support this notion, our bioinformatics work with ChIPBase software [39] identified multiple NF-κB binding sites up- and down-stream of the miR-223 loci in the human X chromosome. Different binding sites used by NF-κB in different cells might cause varying effects on miR-223 expression. Down-regulating HSP70 reduced miR-223 (data not shown), indicating that celastrol-induced HSP70 elevation might play a role in maintaining miR-223 level, through in the later stage after celastrol treatment, as HSP70 elevation needed 6 h.

Finally and interestingly, in addition to miR-223 being affected by NF-κB and mTOR activity and HSP70 levels, miR-223 could regulate these three molecules, providing a clue to understanding the effects of miR-223 on cellular viability. In our system, NF-κB- or mTOR-inhibitor or knocking down HSP70 could reduce viable cells, meaning that these three molecules are all important to survival. However, the effects of NF-κB might dominate if the alterations of these molecules compete, a hypothesis deduced from celastrol’s effects, as this agent resulted in NF-κB inhibition (reducing survival), mTOR inhibition, and HSP70 reduction (both increasing survival), with a final outcome of reduced survival. As such, NF-κB dominance explains why overexpression of miR-223 alone reduced viable cells, even when mTOR activity and HSP70 levels were increased (because such a manipulation inhibited NF-κB activity). That miR-223 down-regulation increased viable cell can also be explained in this way, since it showed the opposite effect on NF-κB, mTOR, and HSP70. Moreover, the observed facts that miR-223 over-expression or down-regulation, when compared with their mock transfections, could reduce or further increase viable cell loss in celastrol-treated samples, were understandable: up- or down-regulating miR-223 could ameliorate or increase celastrol-caused NF-κB and mTOR inhibition, respectively. miR-223 could regulate HSP70 level, yet the effects were inconsistent in two cell lines as well as in situation with and not with celastrol, thus making us difficult to relate miR-223’s effects to its altering HSP70.

## Conclusions

Our work discloses that celastrol, in a NF-κB inhibition/mTOR activation-related way, induces miR-223 in MCF-7 and PC3, and that down-regulating miR-223 could further reduce living cancer cells in samples treated with celastrol. Inhibiting miR-223 induction, in addition to blocking heat shock response [11, 12], is novel way to increase celastrol’s anti-cancer ability.

## Abbreviations

7-AAD: 7-amino-actinomycin D
CFSE: Carboxyfluorescein diacetate succinimidyle ester
DMSO: Dimethyl sulfoxide
FCM: Flow cytometry
HSF-1: Heat shock factor-1
HSP: Heat shock response
HRP: Horseradish peroxidase
mTOR: Mammalian target of rapamycin
miR-223: microRNA-223
NF-κB: Nuclear factor-kappaB
PVDF: Polyvinylidenedifluoride
qRT-PCR: Quantitative real-time PCR
